# Comparison of the glycopattern alterations of mitochondrial proteins in cerebral cortex between rat Alzheimer’s disease and the cerebral ischemia model

**DOI:** 10.1038/srep39948

**Published:** 2017-01-10

**Authors:** Houyou Yu, Changwei Yang, Shi Chen, Yang Huang, Chuanming Liu, Jian Liu, Wen Yin

**Affiliations:** 1 Department of Emergency Medicine, Xijing Hospital, the Fourth Military Medical University, Xi’an, 710032, China

## Abstract

Alzheimer’s disease (AD) and ischemic brain injury are two major neurodegenerative diseases. Mitochondrial dysfunction commonly occurs in AD and ischemic brain injury. Currently, little attention has been paid to the glycans on mitochondrial glycoproteins, which may play vital roles during the process of mitochondrial dysfunction. The aim of this study was to illustrate and compare the glycopattern alterations of mitochondrial glycoproteins extracted from the cerebral cortex of the rat models of these two diseases using High-throughput lectin microarrays. The results shown that the number of lectins with significant differences compared to normal brains was nine for the rat sporadic Alzheimer’s disease (SAD) model and eighteen for the rat middle cerebral artery occlusion (MCAO) model. Interestingly, five lectins showed opposite expression patterns between the SAD and MCAO rat models. We conclude that glycopattern alterations of mitochondrial glycoproteins in the cerebral cortex may provide vital information to help understand mitochondrial dysfunction in AD and ischemic brain injury. In addition, glycans recognized by diverse lectins with opposite expression patterns between these two diseases hints at the different pathomechanisms of mitochondrial dysfunction in AD and ischemic brain injury.

AD is the most common form of dementia with high morbidity among elderly people^1^. This disease is characterized by three hallmark brain lesions, *i*.*e*., extracellular deposition of amyloid β-peptide (Aβ), intracellular neurofibrillary tangles, and progressive loss of neurons and synapses, all of which are particularly found in the hippocampus and cortex of the brain^2^,^3^,^4^. Cerebral ischemia is commonly caused by a transient or permanent reduction in cerebral blood flow, which often leads to ischemic brain injury such as ischemic stroke^5^,^6^. A single stroke can cause dementia by damaging brain regions critical for cognition, while multiple strokes can cause stepwise cognitive deterioration through cumulative brain damage, both of which are driven by the interaction of neurons, glia, vascular cells and matrix components^7^,^8^. The sequelae of a stroke include aphasia, hemiplegia, and dementia, which are associated with ischemia-induced loss of neural function and neuronal death in the affected area^5^,^9^,^10^.

Currently, increasing evidences have strongly demonstrated that there is a close relationship between AD and ischemic stroke^11^,^12^,^13^,^14^. Both of these diseases are neurodegenerative disorders that are marked by progressive impairment of cognitive function^15^,^16^,^17^. Epidemiological investigations have indicated that older people that have had a silent stroke have a higher risk of AD^18^. Clinical studies suggest that AD patients with prior cerebrovascular disease develop dementia faster^19^. In addition, cerebral ischemia can result in an AD-like neuropathology, which includes the increased accumulation of Aβ, abnormal phosphorylation of the tau protein, and up-regulation of apolipoprotein E and presenilin^20^,^21^.

Mitochondria are cellular energy factories that participate in the generation of reactive oxygen species, calcium homeostasis, oxidative stress, and programmed cell death^22^,^23^. Due to the nature of the brain, neuronal cells are susceptible and almost completely reliant on the mitochondria for energy generation, which are crucial in regulating brain function^24^. Many studies have revealed that mitochondrial dysfunction commonly occurs both in AD^25^,^26^,^27^,^28^ and in ischemic brain injury^29^,^30^,^31^,^32^,^33^, and contributes to brain cell death during the development of AD^34^ or after cerebral ischemia^35^. In mitochondria, glucose metabolism is the main source of energy for mammalian brain, in which neurons have the continuous and highest energy demand^36^. Glucose metabolism provides the foundation for neuronal and non-neuronal cellular maintenance^36^. Disturbed glucose metabolism in mitochondria in the neurons commonly leads to the interference of neurotransmitter synthesis and synaptic activity, thus influencing neurotransmission^37^.

Glycosylation is one of the most common and frequent post-translational protein modifications, which occurs in approximately half of proteins^38^,^39^. There are two major types of protein glycosylation, including *N*-glycosylation of the asparagine side-chains and *O*-glycosylation of the serine or threonine side-chains^40^. Glycosylation increases the diversity of proteins due to its different glycosidic bond, glycan structure, glycan composition, and glycan length^40^. Glycoproteins play important roles in cell-cell and cell-matrix interactions, cell adhesion, evasion of the immune response, development and morphogenesis, malignant transformation and metastasis, and other biological functions^41^,^42^. Glycosylation has also been identified in the mitochondria and participates in mitochondrial protein function and localization^43^,^44^.

Abnormal glycan structures, such as incomplete synthesis or neo-synthesis, are frequently associated with disease onset and progression^39^,^42^,^45^. In the past few years, scientific interest in the abnormal expression of the mitochondrial proteome has increased not only for AD^46^ but also for ischemic brain injury^47^. However, little attention has been paid to the glycopattern of mitochondrial proteins in AD and ischemic brain injury, which may possibly be a novel direction for studying the observed mitochondrial dysfunction in neurodegenerative diseases. The aim of this study was to identify and compare the differentially expressed glycopatterns on the mitochondrial glycoproteins from the streptozotocin-induced rat model of sporadic AD and the MCAO rat model of ischemic brain injury using lectin microarrays.

## Materials and Methods

### Animals

Healthy male Sprague-Dawley rats weighing 200–240 g were obtained from the laboratory animal center of the Fourth Military Medical University. These rats were housed under automatically controlled conditions of temperature (24 °C) and humidity (50%), as well as a light-dark cycle of 12:12 hours, and were allowed to diet freely. All experimental protocols for animal use were in accordance with the corresponding guidelines of the Fourth Military Medical University and approved by the ethics committee of the university.

### Streptozotocin-induced sporadic Alzheimer’s disease rat model

The rats were divided into three groups: SAD-C (without treatment), SAD (subject to intracerebroventricular (icv) streptozotocin (STZ) injection), and SAD-S (sham group of SAD). Before surgery, rats were starved for 12 hours, except for drinking water. During surgery, rat body temperature was maintained at 36.5 ± 0.5 °C. First, 15% chloral hydrate (3 ml/kg, Sigma, St. Louis, MO, USA) was used to anesthetize the rats. For the SAD model group, rats received a single icv injection of 3 mg/kg STZ (5 μl, Sigma) using a Hamilton micro-syringe. For the sham group, rats received 5 μl of vehicle solution (citrate buffer). Five weeks after surgery, the rat body weight (g) was measured. Before removing the brain to use, brain weight (g) was measured. The icv injection was performed using the following coordinates: 0.8 mm posterior to the bregma, 1.5 mm lateral to the saggital suture, and 3.6 mm ventral from the brain surface^48^. Postoperatively, rats were housed individually.

### Morris water maze

Four weeks after icv STZ injection, rats were subjected to the Morris water maze to assess their learning and memory ability. Briefly, a circular tank (120 cm in diameter, 60 cm in height) was filled with opaque water (22 ± 2 °C) up to 30 cm. The tank was divided into four equal quadrants (northeast, northwest, southwest, and southeast). Then, a platform (12.5 cm in diameter) was submerged 2.0 cm below the water surface in the center of one of the quadrants for all trials. Four equally spaced points around the edge of the tank were located at the intersection of the quadrants and were used as start positions. Before training, all rats were habituated to water and the apparatus. Then, rats were trained to perform two trials daily for five consecutive days. For each trial, rats were allowed to locate the platform within a maximum time of 60 s. If rats did not finish this process within 60 seconds, they were guided to the platform by the experimenter. After mounting to the platform, rats were allowed to stay on it for 30 s. On the sixth day, the rat was subjected to a probe trial after removing the platform to test their memory within 60 seconds. During the Morris water maze test, a camera was fixed above the tank, and the time of escape latency (seconds) was recorded and analyzed by one-way ANOVA^49^,^50^. During data analysis, LSD test was used for post hoc multiple comparisons.

### Immunohistochemistry assay

Immunohistochemistry was performed according to the previously described method. After incubation with 3% H_2_O_2_ for 10 min, sections were blocked with 10% goat serum at room temperature for 15 min and then incubated with the primary antibodies of rabbit polyclonal anti-rat β-amyloid 1–42 (1:2000, Abcam, Cambridge, MA, USA) or rabbit monoclonal anti-rat phosphorylated-Tau (phospho S396, 1:4000, Abcam) overnight at 4 °C. After washing with PBS for three times, sections were incubated with biotin-conjugated goat anti-rabbit IgG secondary antibody at 37 °C for 15 min. After washing again, sections were incubated with horse radish peroxidase (HRP)-conjugated streptavidin at 37 °C for 15 min. At the end of incubation, sections were stained by 3,3-diaminobenzidine (DAB) chromogen at room temperature for 15 min in the dark. All sections were visualized and photographed under a light microscope (Olympus IX70, Olympus Optical Co. Ltd, Japan).

### Middle cerebral artery occlusion model

A MCAO model was used to produce the cerebral ischemia as reported previously with some modifications^51^. Before surgery, rats were starved for 12 hours, but allowed to drink water freely. Rat body temperature was monitored and maintained at 36.5 ± 0.5 °C during the surgery. After anesthetizing with 15% chloral hydrate (3 ml/kg), the rats were fixed in a supine position. Then, the right carotid sheath of these rats was exposed through a midline neck incision under a dissecting microscope (SXE-1, Shanghai Precision Instrument, Shanghai, China). The common carotid artery, internal carotid artery (ICA) and external carotid artery (ECA) were carefully dissociated. Then, the ECA was coagulated distal to the bifurcation. A 0.24-mm diameter nylon filament (Beijing Cinontech Co. Ltd., Beijing, China) with a round tip (diameter 0.32 ± 0.02 mm) was inserted into the ICA through the ECA stump and then gently advanced 18–19 mm to occlude the origin of the middle cerebral artery (MCA), which was stayed and fixed thus modeling a permanent ischemia. In the sham group, the nylon filament was not advanced to the origin of the MCA. Postoperatively, rats were housed individually. In all, these rats were divided into five groups: MC (without treatment), M16 (subjected to MCAO for 16 h), M16-S (sham group of M16), M48 (subjected to MCAO for 48 h), and M48-S (sham group of M48).

### Neurological scoring for the MCAO model

Neurological deficit scores were performed according to the criteria of Hara *et al*.^52^. Briefly, a score of 3 indicates loss of walking or righting reflex (severe), 2 indicates contralateral circling (moderate), 1 indicates a failure to extend the forepaw (mild), and 0 indicates no deficits (normal). Rat neurological deficit was evaluated after MCAO surgery for 16 or 48 hours in a blinded fashion. For the MCAO groups, scores of less than 2 were excluded. Data were presented as mean ± standard deviation (SD) of 5 rats from each group, and analyzed by Wilcoxon Rank Sum Test.

### Determination of infarct Size by TTC staining of the MCAO model

2,3,5-Triphenyltetrazolium chloride (TTC) is a sensitive histochemical indicator, that can reflect the activity of mitochondrial respiratory enzymes^53^. Cerebral infarct size caused by an ischemic insult is commonly determined using the TTC stain. The mitochondrial function of infarct tissues is irreversibly impaired. After MCAO surgery for 16 or 48 hours, brains were removed and then cut into five slices of 2.0 mm. Finally, the slices were stained with 2% TTC (Sigma) at 37 °C for 30 min and photographed. For M16 and M48 groups, measurement of infarct area was measured on the 5 TTC stained sections with the use of a Global Laboratory Image analysis program (Data Translation, Marl-boro, MA).

### Mitochondrial protein preparation

Since mitochondrial dysfunction or abnormalities were detected in the cerebral cortex of both MCAO and SAD model rats in previous studies^48^, cells in the cerebral cortex were isolated for mitochondria purification. After SAD surgery for five weeks or MCAO surgery for the indicated times (16 or 48 hours), rats in the different groups were anesthetized by chloral hydrate and executed by neck-breaking. Rat total brain tissue was separated gently. The whole cerebral cortex tissue was obtained and kept on ice and hippocampus was isolated. Then, a mitochondrial isolation kit (Cat No.: MITOISO1, Sigma) for animal tissue was used to separate the mitochondria from cells of the cerebral cortex according to the manufacturer’s instructions. Transmission electron microscopy (TEM) was performed to identify the purify of mitochondria. Then, the acquired pellets were lysed with RIPA buffer (Cat No.: R0278, Sigma).

### Protein Cy3 fluorescent labeling

To allow for individual variations and to normalize the differences, five mitochondrial protein samples from each group (SAD-C, SAD, MC, M16 or M48) were pooled and quantified using a BCA assay kit (Cat No.: QPBCA, Sigma) respectively. Proteins were labeled with Cy3 fluorescent dye (Sigma) and purified with Sephadex G-25 columns (Cat No.: G2580, Sigma) as per the product specifications.

### Lectin microarrays

A lectin microarray was produced to determine whether the mitochondrial protein glycopatterns changed in the SAD or MCAO rat models according to a previous protocol^40^,^54^. To be specific, 37 lectins (Table S1; 29 from Vector Laboratories, Burlingame, CA, USA; seven from Sigma; one from Calbiochem, San Diego, CA, USA) that bind *N*- or *O*-linked glycans were used to produce the lectin microarray. Layout of the lectin microarray was shown in Figure S1. Lectins were dissolved to 1 mg/ml buffer containing 1 mM of the appropriate monosaccharide. The dissolved lectins were spotted onto homemade epoxy silane-coated slides with Stealth micro spotting pins (TeleChem, Atlanta, GA, USA) by a Capital smart microarrayer (CapitalBio, Beijing, China). For each lectin, there were three continuous spots per block and three blocks per slide. The slides were put into an incubator with 50% humidity overnight, and then immobilized in a vacuum dryer at 37 °C for 3 hours. After incubation, the slides were blocked with 1 × PBS (0.01 M phosphate buffer containing 0.15 M NaCl, pH7.4) containing 2% (w/v) BSA (Sigma) for 1 hour, washed twice with 1 × PBST (0.2% Tween 20 in 1 × PBS), and then washed once with 1 × PBS. Cy3-labeled protein (4 μg) was diluted in 0.5 ml 1 × PBS containing 2% (w/v) BSA, 500 mM glycine (Sigma) and 0.1% Tween-20 (Sigma), then incubated with the blocked lectin microarray slides in a chamber at 37 °C for 3 hours in a rotisserie oven at 4 rpm. Afterwards, the slides were washed twice with 1 × PBST and once with 1 × PBS, and then centrifuged to dry at 600 rpm.

### Data analysis

The slides were scanned with a Genepix 4000B confocal scanner (Axon Instruments, Foster City, CA, USA). Cy3 fluorescence intensity of the acquired image for each spot was analyzed at 532 nm using Genepix 3.0 software (Axon Instruments Inc., CA, USA). After subtracting the average background value, median of each lectin was globally normalized to the sum of all the medians of each lectin in one block. The normalized medians from nine repeated blocks from three slides were averaged and analyzed using the Student’s *t*-test and SPSS 19 software. *P* > 0.05 was considered as effective data. For each lectin, a fold change of ≥1.5 or ≤0.67 between the model group and the control group indicated a significant up- or down-regulation of a certain glycan, respectively.

## Results

### Characterization of the rat SAD model

**Figure 1:**
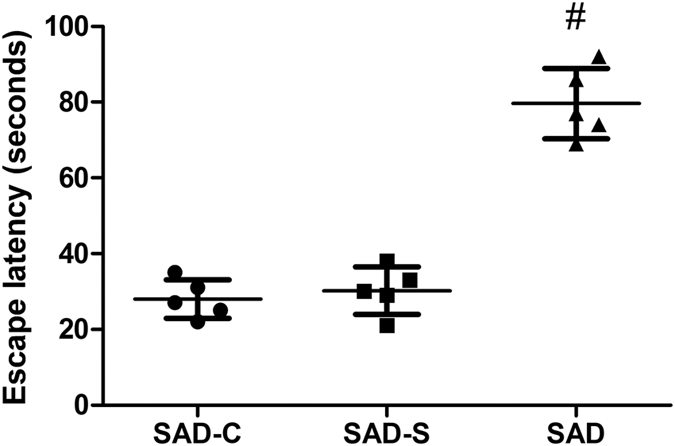
Cognitive performance of rats in the SAD-C, SAD-S and SAD group. The capacity of rats to learn and their memory was assessed by the Morris water maze test 4 weeks after SAD surgery. For the indicated group, each point represents the escape latency of the rat. The data shown are the mean ± SD of five rats in each group. Data were analyzed by one-way ANOVA. ^#^*P* < 0.01 indicates a significant difference when compared with the SAD-C and SAD-S groups.

**Figure 2:**
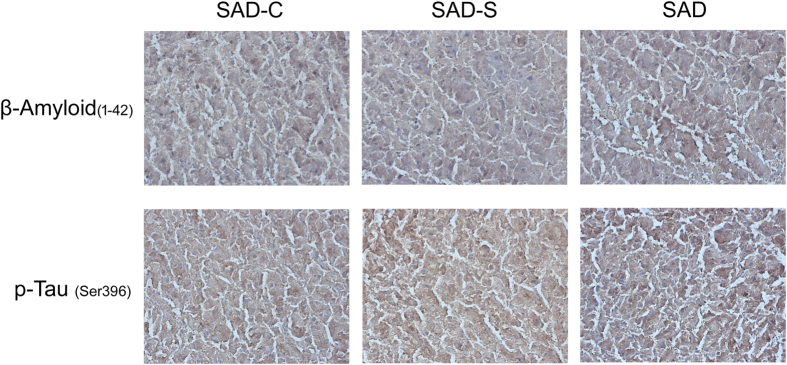
Visualized photographs of deposited β-Amyloid_(1–42)_ or phosphorylated-Tau_(Ser396)_ in cerebral cortex tissues of SAD-C, SAD-S and SAD group. After STZ injection for the indicated time, rat cerebral cortex tissues were acquired and allowed to receive immunohistochemical staining assays as described in methods.

After SAD surgery, the brain and body weight of rats in the SAD-C, SAD-S, and SAD groups were measured, and the brain-to-body weight ratio was calculated. The data shown in Table 1 suggested that both brain weight (g) and the brain-to-body weight ratio in the SAD group were significantly lower than that in the SAD-C or SAD-S groups (*P* < 0.05). This result indicates that the encephalatrophy was only present in the rat SAD model but not in its sham rats. In addition, the Water maze test was performed to evaluate the effectiveness of the rat SAD model. According to the time of escape latency (Fig. 1), rats in the SAD group spent much more time to reach the platform region than rats in the SAD-C or SAD-S groups, which indicates impairment of the cognitive and memory functions after icv STZ injection. In addition, the deposition phenomenon of β-amyloid _(1–42)_ or phosphorylated-Tau _(Ser396)_ protein was more easily to be detected in cerebral cortex tissue of SAD group than that of SAD-C and SAD-S group (Fig. 2). The above results suggest that the rat SAD model was suitable for the following research. Also, based on the above results, rat cerebral cortex mitochondrial proteins in SAD-C and SAD group were separated for lectin microarrays analysis.

### Characterization of the rat MCAO model

**Figure 3:**
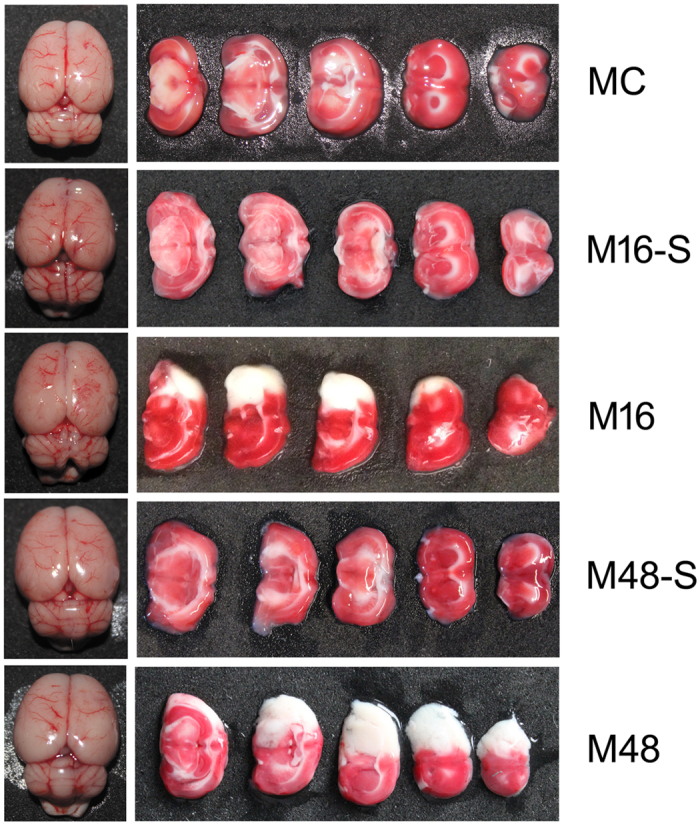
Photographs of rat brains. After MCAO surgery for the indicated time, rat brains were removed and photographed. Then, the brain was cut into slides and subjected to TTC staining. The cerebral infarction in slides was recorded by photographs.

The suitability of the constructed rat MCAO model was evaluated by the neurological score (Table 2), TTC staining (Fig. 3), and infarct volume calculation (Table 3). Neurological deficit occurred after MCAO surgery for both 16 and 48 hours (neurological score > 2), but did not in the sham groups (Table 2). The neurological deficit was more serious with increased ischemia time in the rat MCAO model. From the photographs of the rat brain and the TCC staining (Fig. 3), cerebral infarction appeared only in the right cerebral hemisphere of the rat MCAO model (M16 and M48 group), but not in the MC, M16-S, and M48-S groups. Besides, the total infarct area increased with ischemia time (M16 and M48) in the rat MCAO model (Table 3). These results indicate that the rat MCAO model was suitable for the following research. According to the above results, rats in the MC, M16, and M48 groups were chosen for following experiments.

### Glycopatterns of mitochondrial glycoproteins in the rat SAD model

**Figure 4:**
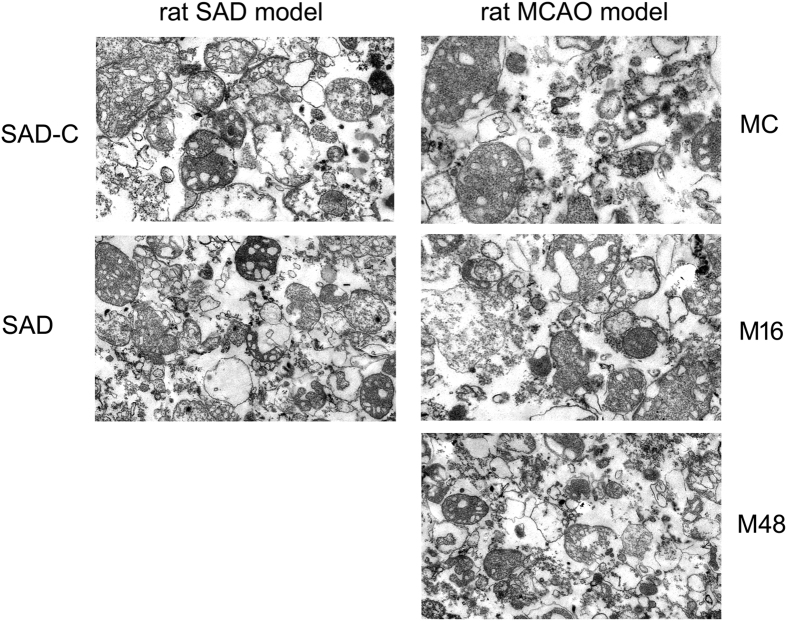
Transmission electron microscopy (TEM) scan graphs of purified mitochondria. After preparation of SAD and MCAO rats, mitochondria were purified from cerebral cortex tissues of SAD-C, SAD, MC, M16, and M48 groups. The purity of mitochondria was proved by TEM.

**Figure 5:**
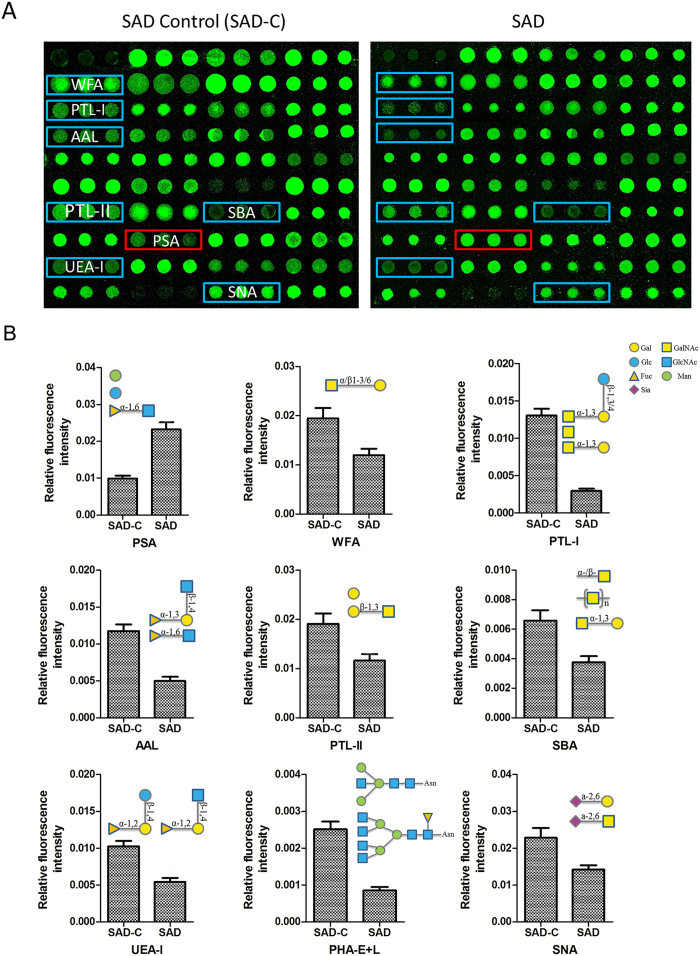
Changes in the glycopatterns of cerebral cortex mitochondrial glycoproteins in the SAD rat model. (**A**) The profile of Cy3-labeled cerebral cortex mitochondrial proteins from the SAD-C and SAD groups bound to lectin microarrays. The fluorescent images were obtained by scanning the microarray with the Genepix 4000B confocal scanner at 70% photomultiplier tube and 100% laser power. For each sample, one of the nine blocks is shown. Lectins marked in a red frame indicate up-regulation, and those marked with a blue frame indicate down-regulation. (**B**) Relative expression levels of glycans recognized by different lectins. Data are presented as mean NFI ± SD of three independent experiments. Significant differences with fold change ≥1.5 or ≤0.67 between groups are shown in Table 4.

The glycopattern of mitochondrial glycoproteins in the cerebral cortex can reflect the expression and function of oligosaccharides in the brain. The aim of this section was to determine whether the glycans on mitochondria glycoproteins were altered in the cerebral cortex of the rat SAD model. Figure 4 reflected that mitochondria samples were collected with high purity and suitable for the following research. Then, the lectin microarray was applied to identify the glycopattern of mitochondrial glycoproteins. The fluorescent images are shown in Fig. 5A. Figure 5B shows the relative fluorescent intensity (normalized fluorescent intensity, NFI) for differential expression of glycans. For these lectins, the NFIs ratio of the SAD to SAD-C groups was calculated, and a ratio ≥1.5 or ≤0.67 represented a significant difference. The NFI of glycans with significant differences are summarized in Table 4. Nine of 37 lectins were significantly altered. Among these, PSA showed markedly up-regulation, while WFA, PTL-I, AAL, PTL-II, SBA, UEA-I, PHA-E + L, and SNA showed marked down-regulation. These results illustrate that the glycopattern of cerebral cortex mitochondrial glycoproteins in the SAD rat is different from that in the normal rat.

### Glycopattern of mitochondria glycoproteins in rat MCAO model

**Figure 6:**
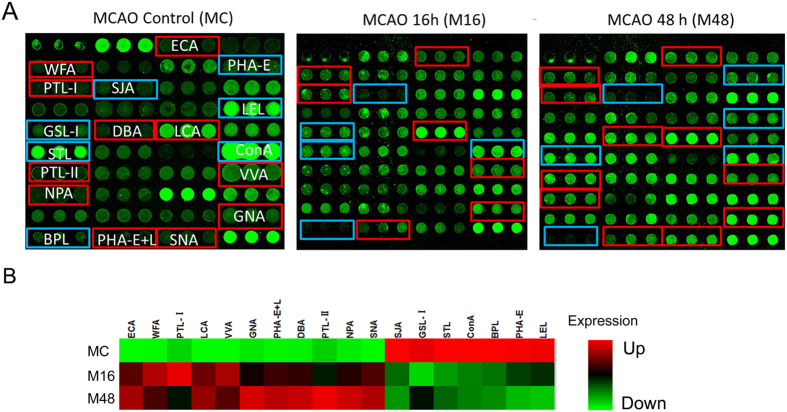
Changes in the glycopattern of cerebral cortex mitochondrial glycoproteins in the MCAO rat model. (**A**) The profile of Cy3-labeled cerebral cortex mitochondria proteins from MC, M16, and M48 groups bound to lectin microarrays. The fluorescent images were obtained by scanning the microarrays with the Genepix 400B confocal scanner. For each sample, one of the nine blocks is shown. Lectins marked with a red frame indicate up-regulation, and those marked with a blue frame indicate down-regulation. (**B**) Heat map and hierarchical clustering analysis of the 18 lectins. Samples are listed in rows and the lectins are listed in columns. The intensity and color of each square indicates expression levels relative to other data in the column. Green indicates low, black indicates medium, while red indicates high.

**Figure 7:**
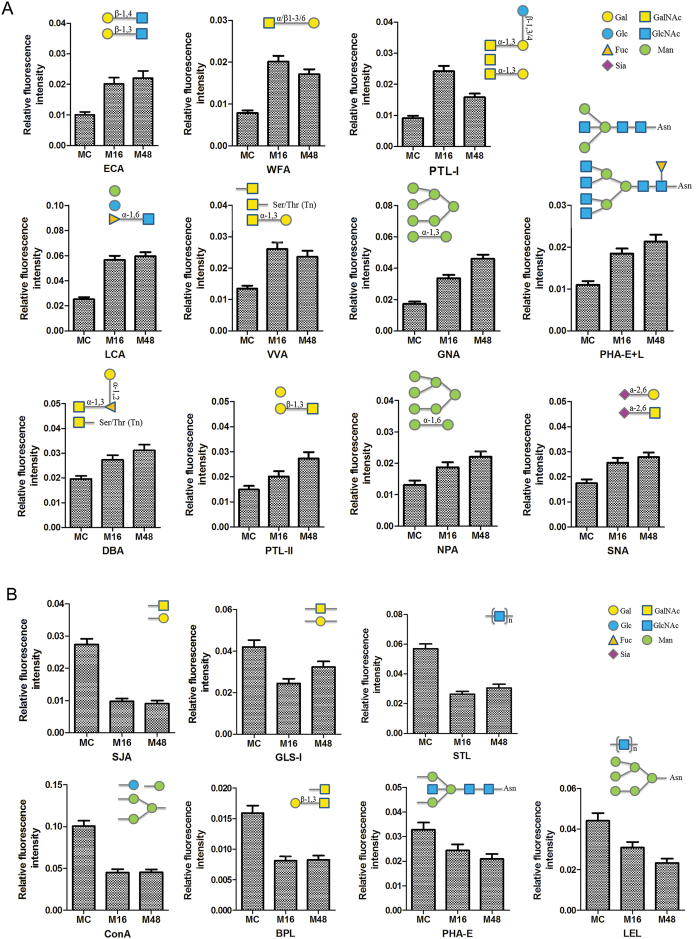
Relative expression levels of glycans recognized by different lectins on cerebral cortex mitochondria glycoproteins in the MCAO rat model. (**A**) Up-regulated glycans. (**B**) Down-regulated glycans. Data are presented as mean NFI ± SD of three independent experiments. Significant differences with fold change ≥1.5 or ≤0.67 between groups are shown in Table 5.

Figure 4 reflected that mitochondria samples were collected with high purity and suitable for the following research. The lectin microarray was applied to identify differentially expressed glycans on cerebral cortex mitochondrial glycoproteins during the process of cerebral ischemia in the rat MCAO model. The fluorescent images for the MC, M16, and M48 groups are shown in Fig. 6A. Figure 6B shows the heat map and hierarchical clustering analysis of the fluorescent intensity for differentially expressed lectins. Figure 7 shows the NFI of differentially expression glycans. For these lectins, the NFI ratios of M16 to MC and M48 to MC were calculated. A ratio ≥1.5 or ≤0.66 represented significant differences. The NFI for glycans with significant differences are summarized in Table 5. Eighteen of 37 lectins showed significant changes in the M16 or M48 group when compared with that in the MC group. Among these lectins, 11 of these 18 lectins showed marked up-regulation, including ECA, WFA, PTL-I, LCA, VVA, GNA, PHA-E + L, DBA, PTL-II, NPA, and SNA. However, DBA, PTL-II, NPA and SNA were up-regulated only in the M48 group. Furthermore, seven of these 18 lectins showed marked down-regulation, including SJA, GLS-I, STL, ConA, BPL, PHA-E, and LEL. However, GLS-I was down-regulated only in the M16 group, while PHA-E and LEL were down-regulated only in the M48 group. These results reveal that the glycopattern of cerebral cortex mitochondria glycoproteins showed greater changes with increasing cerebral ischemia time in the rat MCAO model, which is in contrast to the primary glycopattern of cerebral cortex mitochondrial glycoproteins.

### Lectins with differential expression in both the rat SAD and the MCAO model

According to the above results, in total there are five lectins including WFA, PTL-I, PTL-II, PHA-E + L, and SNA that showed differential expression in both the rat SAD model and the MCAO model. Interestingly, all of these five lectins were markedly down-regulated in the rat SAD model but markedly up-regulated in the rat MCAO model. This distinction may hint at the different pathomechanisms for mitochondrial dysfunction between AD and ischemic brain injury. For these five lectins, the NIF ratios of the SAD to SAD-C group, the M16 to MC group, and the M48 to MC group are summarized in Table 6. Among these lectins, WFA showed the greatest changes in the rat MCAO model, while PTL-I showed the greatest changes in rat SAD model.

## Discussion

There is a close-knit relationship between AD and ischemic stroke^5^. Both AD and ischemic stroke are characterized by a widespread functional disturbance of the human brain^55^, which is associated with progressive loss or damage of neurons^56^. In neurodegenerative diseases, mitochondrial dysfunction has been reported to occur early and to act causally in disease pathogenesis^57^. Furthermore, in multiple diseases, the glycopattern of proteins is different to that in normal samples^39^,^40^,^58^, and the differentially expressed glycans may play an important role in the pathological process. Many studies have focused on the mitochondrial proteome to explore the pathogenesis of AD^46^ and ischemic stroke^47^; however, there has been little focus on the glycopattern of the mitochondrial glycoproteins in these two diseases.

Lectins, which are commonly derived from plants and sometimes invertebrates, can be applied to reflect glycan structures of glycoconjugates, such as glycoproteins, glycolipids and glycosaminoglycans^59^. Lectin microarrays are a novel technology for glycan analysis that can reflect the protein glycopattern in samples without the need for glycan release^60^. In this study, lectin microarrays were used to explore the alteration of mitochondrial protein glycopatterns in the cerebral cortex of the rat SAD model of AD and the MCAO model of ischemic brain injury. According to the results, nine lectins were altered in the rat SAD model (Table 4) and 18 in the rat MCAO model (Table 5). Five lectins (WFA, PTL-I, PTL-II, PHA-E + L, and SNA) showed opposite changes between the SAD and MCAO rat models. The above findings may supply vital clues to help study mitochondrial dysfunction-related pathomechanisms of AD and ischemic brain injury. What’s more, glycans recognized by these lectins in these two diseases should be given much more attention, since they may hint at discrete pathomechanisms of mitochondrial dysfunction in AD and ischemic brain injury.

Without doubt, protein glycosylation plays an important role in the pathogenesis and progression of various diseases^61^,^62^,^63^. In the mitochondria, protein glycosylation participates in mitochondrial protein function and localization^43^. For instance, Anello *et al*. reported that excessive protein *N*-glycosylation of mitochondrial F_1_-F_0_-ATP-synthase led to cell damage and secretory alterations in pancreatic β-cells^44^. Kung, *et al*. discovered that 30 mitochondrial proteins can react with ConA and WGA^43^, among which Lpe10p is a mitochondrial inner-membrane magnesium transporter that is involved in the maintenance of magnesium concentrations inside mitochondria. In our study, we observed effective fluorescence signals for ConA and WGA in all of the mitochondrial protein samples. In addition, mitochondrial glycoproteins can also react with the rest 35 selected lectins, all of which were observed to show an effective fluorescence signal.

Although the lectin microarray cannot identify a certain glycoprotein in samples, it can reflect the entire glycopattern of glycoproteins in different samples. In this study, we found that the glycopattern of mitochondrial glycoproteins in the cerebral cortex of the rat SAD model was different from that in normal rats. The differentially expressed glycans may be closely associated with the mitochondrial function-related onset and progression of AD. Among these nine abnormally expressed lectins, eight lectins were down-regulated. Similarly, the glycopattern of cerebral cortex mitochondrial glycoproteins in the rat MCAO model was also different to that in normal rats. We speculate that these glycan changes are related to the mitochondrial dysfunction in ischemic brain injury. The number of abnormally expressed lectins increased with the time of cerebral ischemia (11 for 16 hours and 18 for 48 hours). The glycopattern of the mitochondrial glycoproteins in the cerebral cortex in the acute cerebral ischemic state changed more intensely than that in AD, and hence, we speculate that the pathomechanism of mitochondrial dysfunction in acute neurodegeneration is different from that in chronic neurodegeneration.

In summary, our study reveals the glycopattern of mitochondrial glycoproteins in the cerebral cortex of the rat AD and cerebral ischemic models. In both models, abnormally expressed glycans that are recognized by different lectins were detected in the cerebral cortex mitochondrial proteins. Five lectins showed opposite changes in the rat SAD and MCAO models. And the common characteristic of glycan structure recognized by these five lectins mainly consists of Gal and GalNAc monosaccharides. Hence, further research should focus on galactosyltransferase or *N*-acetylgalactosaminyltransferase in cerebral cortex cells when comparing the different pathomechanisms of mitochondrial dysfunction between AD and ischemic brain injury. Our research supplies a new direction with which to investigate the mitochondrial dysfunction-related pathomechanism of neurodegeneration.

## Additional Information

**How to cite this article**: Yu, H. *et al*. Comparison of the glycopattern alterations of mitochondrial proteins in cerebral cortex between rat Alzheimer’s disease and the cerebral ischemia model. *Sci. Rep.* **7**, 39948; doi: 10.1038/srep39948 (2017).

**Publisher's note:** Springer Nature remains neutral with regard to jurisdictional claims in published maps and institutional affiliations.

## Supplementary Material

Supplementary Information

## Figures and Tables

**Table 1:** Characterizations of rat SAD model.

Parameters	SAD-C	SAD-S	SAD
Brain weight (g)	1.877 ± 0.044	1.884 ± 0.049	1.621 ± 0.045*
Weight ratio of brain-to-body	0.00456 ± 0.00031	0.00445 ± 0.00033	0.00343 ± 0.00029*

The brain and body weight were measured immediately after the sacrifice of rats. Data represents mean ± SD of 5 rats from each group. *P < 0.05 indicates significant difference when compared with SAD-C and SAD-S group.

**Table 2:** Neurobehavioral scores.

Groups	Number	Scores
MC	5	0 ± 0
M16-S	5	0 ± 0
M16	5	2.13 ± 0.19*
M48-S	5	0 ± 0
M48	5	2.64 ± 0.33*

Neurological deficit was scored by criteria described in methods after MCAO surgery for 16 or 48 h. Data represents mean ± SD of 5 rats from each group, and analyzed by Wilcoxon Rank Sum Test. For M16 group, *P < 0.05 indicates significant difference when compared with MC or M16-S group. For M48 group, *P < 0.05 indicates significant difference when compared with MC or M48-S group.

**Table 3:** Total infarct area of brain tissue of MCAO model.

Groups	Number	Percentage of total infarct area (%)
M16	5	35.26 ± 4.17
M48	5	62.85 ± 5.94*

The percentage of total infarct area of brain tissue of M16 or M48 group was caculated by protocols described in methods. Data represents mean ± SD of 5 rats from each group. *P < 0.05 indicates significant difference when compared with M16 group.

**Table 4:** Changes in the Glycopattern of Mitochondria Glycoproteins in Cerebral Cortex of SAD rat model.

Lectins	Specificity Glycans	Ratio (SAD/SAD-C)
PSA	Fucα-1,6GlcNAc, α-D-Man, α-D-Glc	2.35
WFA	terminating in GalNAcα/β1-3/6 Gal	0.61
PTL-I	GalNAc, GalNAcα-1,3 Gal, GalNAcα-1,3Galβ-1,3/4Glc	0.22
AAL	Fucα1-6 GlcNAc(core fucose), Fucα1-3(Galβ1-4)GlcNAc	0.42
PTL-II	Gal, blood group H, T-antigen	0.61
SBA	α- or β-linked terminal GalNAc, (GalNAc)n, GalNAcα1-3 Gal	0.57
UEA-I	Fucα1-2Galβ1-4Glc(NAc)	0.53
PHA-E + L	Bisecting GlcNAc, bi-antennary N-glycans tri- and tetra-antennary complex-type N-glycan	0.34
SNA	Sia2-6Gal/GalNAc	0.62

Changes were identified by lectin microarray analysis based on data of 9 lectins giving significant differences (Fold change ≥ 1.5 or ≤0.67). The NFIs of each lectin from this two groups were compared based upon fold-changes.

**Table 5:** Changes in the Glycopattern of Mitochondria Glycoproteins in Cerebral Cortex of MCAO rat model.

Lectins	Specificity Glycans	Ratio	
		M16/MC	M48/MC
ECA	Galβ-1,4GlcNAc (type II), Galβ1-3GlcNAc (type I)	1.99	2.20
WFA	terminating in GalNAcα/β1-3/6 Gal	2.54	2.17
PTL-I	GalNAc, GalNAcα-1,3 Gal, GalNAcα-1,3Galβ-1,3/4Glc	2.66	1.74
LCA	α-D-Man, Fucα-1,6GlcNAc, α-D-Glc	2.24	2.37
VVA	terminal GalNAc, GalNAcα-Ser/Thr(Tn), GalNAcα1-3 Gal	1.93	1.75
GNA	High-Mannose, Manα1-3Man	1.94	2.67
PHA-E + L	Bisecting GlcNAc, bi-antennary N-glycans, tri- and tetra-antennary complex-type N-glycan	1.68	1.94
DBA	αGalNAc, Tn antigen, GalNAcα1-3((Fucα1-2))Gal (blood group A antigen)	1.39	1.58
PTL-II	Gal, blood group H, T-antigen	1.35	1.84
NPA	High-Mannose, Manα1-6Man	1.43	1.69
SNA	Sia2-6Gal/GalNAc	1.47	1.60
SJA	Terminal in GalNAc and Gal	0.40	0.33
GLS-I	αGalNAc, αGal, anti-A and B	0.58	0.77
STL	trimers and tetramers of GlcNAc, core (GlcNAc) of N-glycan, oligosaccharidecontaining GlcNAc and MurNAc	0.46	0.54
ConA	High-Mannose, Manα1-6(Manα1-3)Man, αMannose, αGlc	0.45	0.45
BPL	Galβ1-3GalNAc, Terminal GalNAc	0.55	0.52
PHA-E	Bisecting GlcNAc, biantennary complex-type N-glycan with outer Gal	0.74	0.64
LEL	(GlcNAc)n, high mannose-type N-glycans	0.70	0.53

Changes were identified by lectin microarray analysis based on data of 18 lectins giving significant differences (Fold change ≥1.5 or ≤0.67). The NFIs of each lectin from this three groups were compared based upon fold-changes.

**Table 6:** Lectins with differential expression both in rat SAD and MCAO model.

Lectins	Ratio		
	SAD/SAD-C	M16/MC	M48/MC
WFA	0.61	2.54	2.17
PTL-I	0.22	2.66	1.74
PTL-II	0.61	1.35	1.84
PHA-E + L	0.34	1.68	1.94
SNA	0.62	1.47	1.60

Changes were identified by lectin microarray analysis based on data of 5 lectins giving significant differences both in rat SAD and MCAO model (Fold change ≥1.5 or ≤0.67). Fold changes of NFIs between corresponding groups were shown.
