# Supplementary material for: Comparison of the glycopattern alterations of mitochondrial proteins in cerebral cortex between rat Alzheimer’s disease and the cerebral ischemia model
Source: Sci Rep. 2017 Jan 10;7:39948. doi: 10.1038/srep39948 (PMC5223200; doi:10.1038/srep39948)
Supplement: Supplementary Information [file srep39948-s1.doc]

**Comparison of the glycopattern alterations of mitochondrial proteins** **in cerebral cortex between rat** [**Alzheimer's**](javascript:void(0);) [**disease**](javascript:void(0);) **and the cerebral ischemia model**

**Houyou Yu****^†^, Changwei Yang^†^, Shi Chen, Yang Huang, Chuanming Liu, Jian Liu, and** **Wen Yin***

Department of Emergency Medicine, Xijing Hospital, Fourth Military Medical University, Xi’an, 710032, China

*Corresponding author, Wen Yin. Email: [wenyinxian029@163.com](mailto:wenyinxian029@163.com)

†These authors contributed equally to this work.

Running title: Comparison of glycopattern alterations of mitochondrial proteins between rat AD and MCAO model

**Supplementary Figures and Tables:**


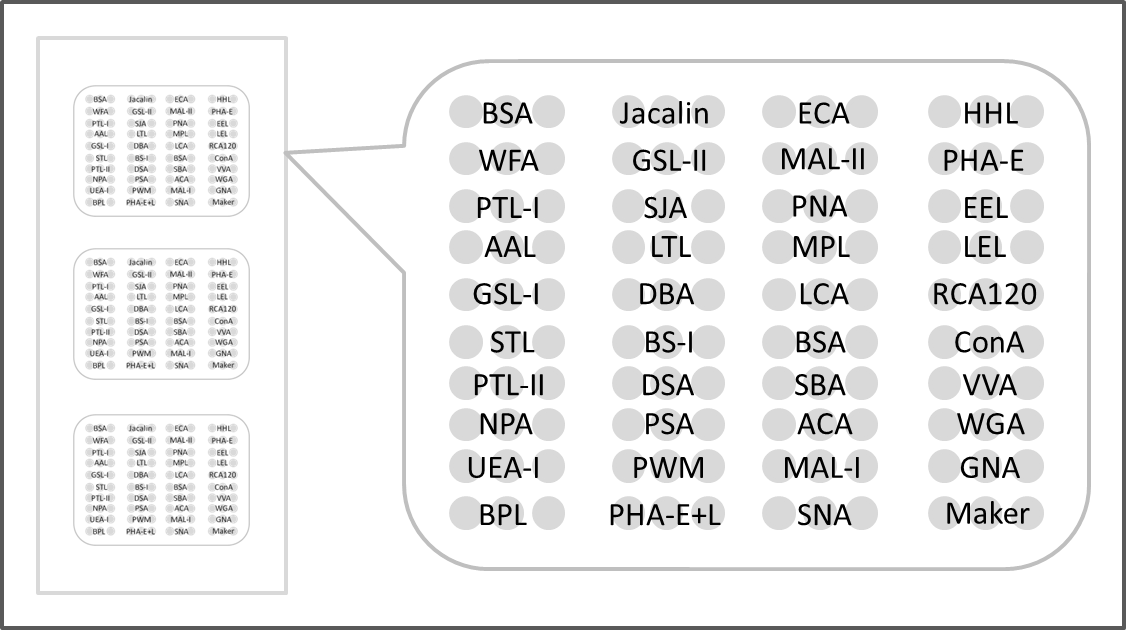


**Figure S1. Layout of the lectin microarray.** Information for lectins are shown in Table S1.

Table S1 Glycan-binding speciﬁcities of lectins

| **Lectin** | **Specificity Glycans** | **Print**  **monosaccharide** | **Supplier** |
| --- | --- | --- | --- |
| Jacalin | Galβ1-3GalNAcα-Ser/Thr(T),GalNAcα-Ser/Thr(Tn), GlcNAcβ1-3- GalNAcα-Ser/Thr(Core3), sialyl-T(ST). not bind to Core2, Core6, and sialyl-Tn (STn) | Galactose | Vector |
| ECA | Galβ-1,4GlcNAc (type II), Galβ1-3GlcNAc (type I) | Galactose | Vector |
| HHL | High-Mannose, Manα1-3Man, Manα1-6Man, Man5-GlcNAc2-Asn | Mannose | Vector |
| WFA | terminating in GalNAcα/β1-3/6Gal | GalNAc | Vector |
| GSL-II | GlcNAc and agalactosylated tri/tetra antennary glycans | GlcNAc | Vector |
| MAL-II | Siaα2-3Galβ1-4Glc(NAc)/Glc, Siaα2-3Gal, Siaα2-3, Siaα2-3GalNAc |  | Vector |
| PHA-E | Bisecting GlcNAc, biantennary complex-type N-glycan with outer Gal | GlcNAc | Vector |
| PTL-I | GalNAc, GalNAcα-1,3Gal, GalNAcα-1,3Galβ-1,3/4Glc | GalNAc | Vector |
| SJA | Terminal in GalNAc and Gal, anti-A and anti-B human blood group | GalNAc | Vector |
| PNA | Galβ1-3GalNAcα-Ser/Thr(T) | Galactose | Vector |
| EEL | Galα1-3(Fucα1-2)Gal (blood group B antigen) | Galactose | Vector |
| AAL | Fucα1-6 GlcNAc(core fucose), Fucα1-3(Galβ1-4)GlcNAc | Fucose | Vector |
| LTL | Fucα1-3Galβ1-4GlcNAc, Fucα1-anti-H blood group specificity | Fucose | Vector |
| MPL | Galβ1-3GalNAc, GalNAc | GalNAc | Vector |
| LEL | (GlcNAc)n, high mannose-type N-glycans | LacNAc | Vector |
| GSL-I | αGalNAc, αGal, anti-A and B | GalNAc | Vector |
| DBA | αGalNAc, Tn antigen, GalNAcα1-3((Fucα1-2))Gal (blood group A antigen) | GalNAc | Vector |
| LCA | α-D-Man, Fucα-1,6GlcNAc, α-D-Glc | Mannose | Vector |
| STL | trimers and tetramers of GlcNAc, core (GlcNAc) of N-glycan, oligosaccharide containing GlcNAc and MurNAc | GlcNAc | Vector |
| PTL-II | Gal, blood group H , T-antigen | Galactose | Vector |
| DSA | (GlcNAc) 2-4, polyLacNAc and LacNAc (NA3, NA4) | GlcNAc | Vector |
| VVA | terminal GalNAc, GalNAcα-Ser/Thr(Tn), GalNAcα1-3Gal | GalNAc | Vector |
| MAL-I | Galβ-1,4GlcNAc | Galactose | Vector |
| GNA | High-Mannose, Manα1-3Man | Mannose | Vector |
| NPA | High-Mannose, Manα1-6Man | Mannose | Vector |
| ACA | Galβ1-3GalNAcα-Ser/Thr (T antigen), sialyl-T(ST) tissue staining patterns are markedly different than those obtained with either PNA or Jacalin | Galactose | Vector |
| BPL | Galβ1-3GalNAc, Terminal GalNAc | Galactose | Vector |
| PHA-E+L | Bisecting GlcNAc, bi-antennary N-glycans, tri- and tetra-antennary complex-type N-glycan | GlcNAc | Vector |
| SNA | Sia2-6Gal/GalNAc | GlcNAc | Vector |
| RCA120 | β-Gal, Galβ-1,4GlcNAc (type II), Galβ1-3GlcNAc (type I) | Galactose | Sigma |
| BS-I | α-Gal, α-GalNAc, Galα-1,3Gal, Galα-1,6Glc | Galactose | Sigma |
| PSA | Fucα-1,6GlcNAc, α-D-Man, α-D-Glc | Fucose | Sigma |
| SBA | α- or β-linked terminal GalNAc, (GalNAc)n, GalNAcα1-3Gal, blood-group A | GalNAc | Sigma |
| WGA | Multivalent Sia and (GlcNAc)_n_ | GlcNAc | Sigma |
| UEA-I | Fucα1-2Galβ1-4Glc(NAc) | Fucose | Sigma |
| PWM | (GlcNAc)_n_ and polyLacNAc | GlcNAc | Sigma |
| ConA | High-Mannose, Manα1-6(Manα1-3)Man, αMannose, αGlc | Mannose | Calbiochem |
